# Recommendations for Improving the Quality of Rare Disease Registries

**DOI:** 10.3390/ijerph15081644

**Published:** 2018-08-03

**Authors:** Yllka Kodra, Jérôme Weinbach, Manuel Posada-de-la-Paz, Alessio Coi, S. Lydie Lemonnier, David van Enckevort, Marco Roos, Annika Jacobsen, Ronald Cornet, S. Faisal Ahmed, Virginie Bros-Facer, Veronica Popa, Marieke Van Meel, Daniel Renault, Rainald von Gizycki, Michele Santoro, Paul Landais, Paola Torreri, Claudio Carta, Deborah Mascalzoni, Sabina Gainotti, Estrella Lopez, Anna Ambrosini, Heimo Müller, Robert Reis, Fabrizio Bianchi, Yaffa R. Rubinstein, Hanns Lochmüller, Domenica Taruscio

**Affiliations:** 1 National Centre for Rare Diseases, Istituto Superiore di Sanità, 00162 Rome, Italy; paola.torreri@iss.it (P.T.); claudio.carta@iss.it (C.C.); domenica.taruscio@iss.it (D.T.); 2 RaDiCo, (The French National Programme on Rare Disease Cohorts), Inserm-UMR S933, National Institute of Health and Medical Research, Hôpital Trousseau, 75018 Paris, France; jerome.weinbach@radico.fr (J.W.); paul.landais@umontpellier.fr (P.L.); 3 Institute of Rare Diseases Research, ISCIII, RDR and CIBERER, 28029 Madrid, Spain; mposada@isciii.es (M.P.-d.-d.-P.); elopez@isciii.es (E.L.); 4 Institute of Clinical Physiology, National Research Council, 56124 Pisa, Italy; alessio.coi@ifc.cnr.it (A.C.); msantoro@ifc.cnr.it (M.S.); fabrizio.bianchi@ifc.cnr.it (F.B.); 5 Fondazione Toscana “Gabriele Monasterio” (FTGM), 56124 Pisa, Italy; 6 Patient Advisory Council of RD Connect and Vaincre la Mucoviscidose the French Cystic Fibrosis Patient Organization, 75013 Paris, France; llemonnier@vaincrelamuco.org; 7 Department of Genetics, University Medical Centre Groningen (UMCG), University of Groningen, 9700 RB Groningen, The Netherlands; d.van.enckevort@rug.nl; 8 Leiden University Medical Center, 2333 ZA Leiden, The Netherlands; m.roos@lumc.nl (M.R.); annika.jacobsen.86@gmail.com (A.J.); 9 Amsterdam UMC, University of Amsterdam, Medical Informatics, Amsterdam Public Health Research Institute, 1105 AZ Amsterdam, The Netherlands; r.cornet@amc.uva.nl; 10 Office for Rare Conditions, Royal Hospital for Children, University of Glasgow, Glasgow G51 4TF, UK; faisal.ahmed@glasgow.ac.uk; 11 Patient Advisory Council of RD-Connect and EURORDIS-Rare Diseases Europe, 75014 Paris, France; virginie.bros-facer@eurordis.org; 12 Patient Advisory Council of RD Connect and MCT8-AHDS Foundation, Oklahoma, OK 74464, USA; veronica_maria_popa@yahoo.com; 13 Patient Advisory Council of RD Connect and NephcEurope Foundation, 2411 DW Bodegraven, The Netherlands; mvanmeel@casema.nl; 14 Patient Advisory Council of RD Connect and FEDERG, 1200 Brussels, Belgium; daniel.renault34@orange.fr; 15 Patient Advisory Council of RD Connect and PRO RETINA Deutschland, 53113 Bonn, Germany; rainald.vongizycki@charite.de; 16 EA2415 Clinical Research Institute, Montpellier University, 34093 Montpellier, France; 17 Department of Public Health and Caring Sciences, Centre for Research Ethics & Bioethics (CRB) Uppsala University, 75122 Uppsala, Sweden; deborah.mascalzoni@crb.uu.se; 18 Bioethics Unit, Office of the President, Istituto Superiore di Sanità, 00162 Rome, Italy; sabina.gainotti@iss.it; 19 Fondazione Telethon, 20129 Milan, Italy; aambrosini@telethon.it; 20 Diagnostic and Research Center for Molecular BioMedicine, Medical University of Graz, 8010 Graz, Austria; heimo.mueller@medunigraz.at (H.M.); robert.reihs@medunigraz.at (R.R.); 21 National Library of Medicine/National Institutes of Health, Bethesda, MD 20892-2128, USA; yaffa.rubinstein@nih.gov; 22 Department of Neuropediatrics and Muscle Disorders Medical Center, University of Freiburg Faculty of Medicine, 79160 Freiburg, Germany; hanns.lochmuller@gmail.com; 23 CNAG-CRG, Centre for Genomic Regulation (CRG), Barcelona Institute of Science and Technology (BIST), 08028 Barcelona, Spain

**Keywords:** rare diseases, patient registry, quality

## Abstract

Rare diseases (RD) patient registries are powerful instruments that help develop clinical research, facilitate the planning of appropriate clinical trials, improve patient care, and support healthcare management. They constitute a key information system that supports the activities of European Reference Networks (ERNs) on rare diseases. A rapid proliferation of RD registries has occurred during the last years and there is a need to develop guidance for the minimum requirements, recommendations and standards necessary to maintain a high-quality registry. In response to these heterogeneities, in the framework of RD-Connect, a European platform connecting databases, registries, biobanks and clinical bioinformatics for rare disease research, we report on a list of recommendations, developed by a group of experts, including members of patient organizations, to be used as a framework for improving the quality of RD registries. This list includes aspects of governance, Findable, Accessible, Interoperable and Reusable (FAIR) data and information, infrastructure, documentation, training, and quality audit. The list is intended to be used by established as well as new RD registries. Further work includes the development of a toolkit to enable continuous assessment and improvement of their organizational and data quality.

## 1. Introduction

In the field of rare diseases (RD), patient registries are a powerful tool that helps develop clinical research, facilitates the planning of appropriate clinical trials, improves patient care, and supports healthcare management. The importance of RD registries is demonstrated by European Union recognition in the document, “EU Council Recommendation of 8 June 2009 on an action in the field of rare diseases” [1]. Patient registries constitute key information systems that support the forthcoming activities of European Reference Networks (ERNs) on rare diseases. In fact, according to Directive 2011/24/EU, a primary aim of ERNs is to “reinforce research (and) epidemiological surveillance like registries” [2].

A rapid proliferation of RD registries has occurred in recent years and according to Orphanet (European website providing information about orphan drugs and rare diseases) there are more than 747 RD registries in Europe [3]. Their objectives are extremely diverse, ranging from clinical patient data management to epidemiology and research goals; each of them are supported by a wide variety of information systems, ontological standards, data collection and management tools, as well as governance models.

In response to these heterogeneities, in the framework of RD-Connect project [4], the National Center of Rare Diseases in Italy coordinated and selected a group of subject matter experts, including members of patient organizations, with wide experience in the field of RD registries. The objective of the expert working group was to make a list of recommendations to be used as a framework for improving the quality of RD registries.

After reviewing several guidelines on RD registries elaborated by EPIRARE (European Platform for Rare Disease Registries) [5], PARENT Joint Action [6], Neurological Registry Best Practice Guidelines in Canada [7] and the Agency for Healthcare Research and Quality (AHRQ) [8], the first draft of this paper elaborated by the Italian National Center of Rare Diseases was presented to the experts and then discussed with them. The experts were asked to provide their comments on the basis of their skills and knowledge on specific parts of the article. The experts’ responses were then collected.

**Figure 1:**
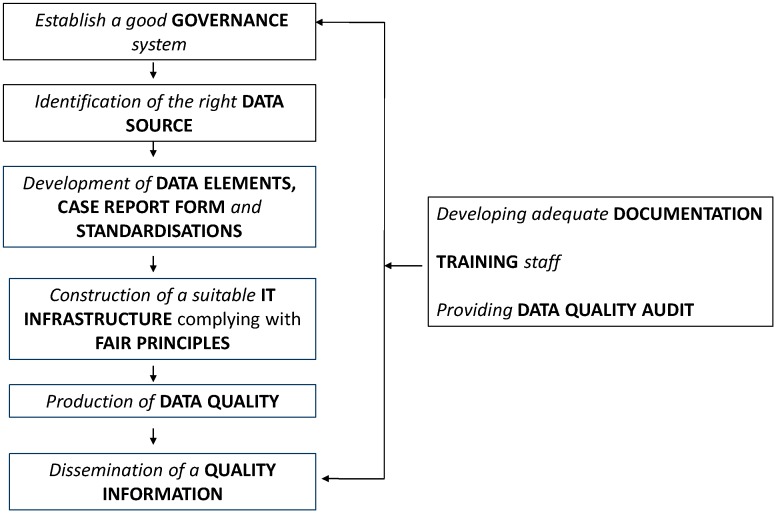
A framework for quality management of RD registries.

As shown in Figure 1, the quality of registries is a global concern and involves all sequential activities for running a registry: (1) Governance; (2) data source; (3) data elements, case report form, standardisations; (4) IT infrastructure complying with FAIR principles (Findable, Accessible, Interoperable, Reusable for humans and computers); (5) data quality; (6) quality information; (7) documentation (8) training staff; (9) and data quality audit.

Recommendations laid out here are developed for each topic and each of them is preceded by a justifying comment.

Before elaborating on these topics in detail, we will first provide definition and classification of registry.

### 1.1. Definition of a Registry

A clear definition of a registry is central to the process of evaluating registries.

In the field of health, several definitions have been provided and a variation in the definition is found over time.

In 1974, Brooke defined registry as a file of documents containing uniform information about individual persons, collected in a systematic and comprehensive way, in order to serve a predetermined purpose [9]. In 1991, another broad definition was provided by Solomon et al., who defined a registry as a database of identifiable persons containing a clearly defined set of health and demographic data, collected for a specific public health purpose [10].

In 2014, the registry definition evolved and it was no longer conceived as a file or database but as an organised system, highlighting the prospectic characteristic of its design. In fact, the most complete definition was provided by the Agency for Healthcare Research and Quality (AHRQ) as: «[…] *a patient registry is an organized system that uses observational study methods to collect uniform data (clinical and other) to evaluate specified outcomes for a population defined by a particular disease, condition, or exposure, and that serves one or more predetermined scientific, clinical, or policy purposes*» [8]. A similar definition was provided by the European Medicines Agency (EMA), playing an important role in pharmacovigilance and providing an adequate source of post-authorisation data for regulatory decision-making.

*Recommendation #1: RD registries are an organised information system, based on observational study with one or several predefined purposes and rules with a long-term perspective. We recommend that RD registries not be limited by geographic area, but have an international vision. In the field of RD, there is a need to identify as many patients as possible, and a significant number of cases can be identified from a large population. Moreover, RD registries should be created and used as a data source for the purpose of performing subsequent or additional studies related to research, patient care, or public health monitoring.*

### 1.2. Registry Classification

Registries can be classified in different ways in accordance with their objectives. They can be divided into three categories with multiple subcategories and combinations: (1) Public health registry (e.g., disease occurrence: estimate incidence and prevalence, temporal trends and geographical distribution in relation to person, place, time); (2) clinical registry (e.g., the study of natural history of disease); (3) product registry (e.g., medical devices or pharmaceutical products) and a combination (e.g., aspects that belong to more than one registry type) [11,12].

Based on geographic coverage, registries can be classified as: population-based registries, which refer to a geographically defined population and their aim is to register all cases in that population; and non-population-based registries, which are based on selected bodies, clinical centres or other types of structures where the population coverage may not be comprehensive [13].

Depending on which disease is under study, a registry can be classified specifically for one disease (e.g., Neurofibromatosis Registry) or group of diseases (e.g., Registry for Neuromuscular Disease).

Registries are also classified according to the type of drivers: (1) patient-driven or patient self-reported (only patients provide information); (2) physician-driven or professional-reported (only physicians provide information) or (3) combination of patient self-report and professional-reported (information is provided by both patients and their physicians).

In addition, based on the method used for data collection, registries are classified as manual/paper-based and/or electronic-based. In electronic registries, part of the registry data can be electronically imported from other data source systems (e.g., omics data from diagnostic laboratories, or data from the patient’s Electronic Health Records and, of growing importance, data from connected devices and smartphone applications) with semi-automatically quality controlled via pre-set rules, avoiding possible manual transcription errors and lowering the burden of manual data entry.

Another category is administrative registry, which collects information for administrative purposes (e.g., national registry of hospital admission, national registry of causes of death). They usually contain information from a large range of conditions. This is in contrast with disease-specific registries, which focus on a single disease or on related groups of diseases and have more clinical background.

Recently, a new registry category, called registry-based randomized controlled trials, has been included [14]. The advantages of registry based randomized controlled trials include low cost, enhanced generalizability of findings, rapid consecutive enrolment, and the potential completeness of follow-up for the reference population [15].

*Recommendation #2: In order to fulfill expected objectives, the taxonomic position of the registry should be clearly defined. It can be classified into several categories according to different criteria (e.g., geographic coverage, diseases coverage, type of sponsors, methods used for data collection, etc.).*

The next subchapters present recommendations for each topics included in Figure 1.

## 2. Governance

The AHRQ guide [8] defines governance as referring to guidance and high-level decision-making, including the following key functions: Defining objectives, identifying stakeholders, building the team, Ethical Legal and Societal Issue (ELSI) and privacy, ensuring sustainability.

### 2.1. Defining Objectives

A registry should have one main general objective associated with specific secondary objectives. The objectives need to be specific, sufficiently detailed, and attainable. Clear objectives are essential to define items to be collected, which patients to include, which ethical and regulatory pathways to go through, which design of the study to opt for, which analytical and reporting approaches to prepare. This will ensure that the registry effectively addresses important scientific questions and collects data that fulfil the precise goal. Attempts to be all inclusive with unclear initial objectives may add cost but no value, resulting in overly data collection that reduces quality and completeness and finally does not allow to adequately address important questions. On the other hand, a good registry should be conceived as agile enough in its intrinsic design to allow addressing new, emerging questions whenever needed by allowing new data items (or specific forms) to be added over time for specific secondary research projects and specific registry patient subgroups. This agility will support its overall sustainability strategy.

*Recommendation #3: Define clear objectives and consequently design the structure of the database. Often, registries start with a single purpose, but can evolve into multiple purposes, addressing the interests of other collaborators and stakeholders. In RD registries, the objectives could be expanded, although they must be prioritized and clearly defined.*

### 2.2. Identifying Key Stakeholders Including the Particular Engagement of Patient Representatives

Identification and early engagement of relevant stakeholders is key to success of a patient registry. Stakeholders include clinicians, patients, family and patient organisations, researchers, and public authorities (e.g., drug agencies), registry funders or private stakeholders (e.g., drug companies, foundations, etc.). The inclusion of more stakeholders increases benefits, but can potentially also complicate decision-making processes. Stakeholders can have conflicting interests and an essential time-consuming step is aligning the aims of the registry with these interests. It is therefore important to determine the main objectives and the expected respective contributions with key stakeholders at an early stage. A key group of stakeholders to consider in the registry governance is patient representatives. Their inclusion can increased enrolment, collection of non-clinical data such as patient reported outcomes, sharing of the results with other patients and provision of financial support. Early inclusion enhances the right of ownership, which also changes their role from passive (patient is a data subject) to active (patient runs the registry). Indeed, several registries for specific RD have been initiated by patient groups. Furthermore, the active engagement of patients at all levels of development ensure that RD registries represent patient needs, increase awareness among all stakeholders of the existence of the registry and, ultimately, improve the quality and quantity of data collected through a patient-centered approach [16]. A variety of methods exist on how to consult with patients and professionals. In the context of the registry, the Dialogue Model appeared feasible to structure the process of collaboration between stakeholders and patient organizations [17].

*Recommendation #4: Engage with all relevant stakeholders, especially patients’ representatives at an early stage in the implementation of a registry.*

### 2.3. Building the Team

A key strength of the registry is that the staff are required to have different expertise for each aspect of the registry system which may include a registry leader (with roles such as strategy, relations with the participating institutions’ administration, financial sustainability, and collaborations); project managers (with roles such as timelines, milestones, deliverables, reports, communication and budgets); domain experts or disease clinical experts (with roles such as content of the registry, selecting appropriate disease variables, endpoints and scoring standards); experts in epidemiology and biostatistics (with roles such as conception, methods, analysis, interpretation and modeling); database managers (with roles such as collection, registration, data management and monitoring); IT personnel (with roles such as information system architects, software developers, back office/helpdesk and bug resolutions, automation and output and ensure technical interoperability); Ethical Legal and Social Issue (ELSI) and privacy specialists (with roles such as ensure compliance with legal requirements and data protection, informed consent); quality assurance experts (with roles such as data quality and quality assurance of procedures); legal affair specialists (partnership contract with private companies, access and IP protection and exploitation issues, consortium agreement, confidentiality agreements, etc.) and administration (e.g., secretarial support). Authorized representatives of the legal entities involved in the registry should also be involved in decision making process and informed about strategic development perspectives as often as possible.

Independent of the size and purpose of the registry, all the expertise described above is required. However, the team size (number of employers) might differ from case to case; one employer might cover more than one expertise; it could be internal or external to the day-to-day operation of the registry.

*Recommendation #5: Establish a good registry team with clear role and responsibilities for all staff working members in proportion with the registry’s size, ambitions, and objectives. Identify a registry leader, domain expert, IT expert and ELSI expert at the start. Either employ an IT person within your registry or setup a long-term partnership with a commercial company.*

### 2.4. Ethical, Legal and Societal Issue (ELSI) and Privacy

Building a solid framework that addresses Ethical, Legal and Social Issues, along with legal requirements, including privacy, is a fundamental cornerstone to build a sustainable future-oriented registry; unfortunately, registry holders are often unfamiliar with these concepts [18].

A registry should be developed in accordance with ELSI principles and rules and in accordance with the main international applicable ethical guidelines and soft regulations, and appropriate professional guidelines (e.g., WMA Helsinki declaration 2013, WMA Declaration of Taipei 2016, Cioms guidelines, Oviedo convention, etc.), even in the absence of explicit legal obligations. ELSI principles arise from ethical and legal considerations to pursue medical research in a responsible and respectful way, including inter alia international and local data protection regulations. Registry owners must be aware of these regulations, but also be conscious that basic principles such as respect for the individual, for ethical and legal issues, and obligation to informed consent are not optional (unless there is a national legal basis to allow collection of data). Complying with international ethical and legal standards is essential to make the registry usable on a larger scale. Consequently, helping data providers identify relevant ethical and legal requirements and how these can be addressed is an essential, but frequently neglected, step when removing possible hurdles to data and sample sharing in the life sciences [19,20]. The most important European law for patient registries is ‘The General Data Protection Regulation (GDPR)’ [21] that seeks to protect individuals with regard to processing of personal data and free movement of such data. The regulation, adopted on 27 April 2016, was officially applied across Europe on 25 May 2018. It replaces the current 1995 data protection directive 95/46/EC. The GDPR will ensure compliance with local legal requirements within that country or region; approval should also be obtained from a medical-ethical committee.

Safeguards for patients are not only based on privacy regulations, but also on informed consent and/or expressed by an ad hoc national law for registries. As already mentioned, consent is not only a legal requirement in most countries, but is traditionally a mean to inform patients and build a trustworthy relationship with them, along with inclusion in the planning phase and in the governance structure of the registries. When informed consent is used, the patient should receive information that explains the purpose of the registry, what information will be stored, how the data will be used, the governance of access to the data by third parties and the foreseen access rules, how the patient can access any data, and how the patient can revoke consent. Consent is often required for publications and for international projects [22], and there is a need for improving the informed consent process in international collaborative rare disease research—effective consent for effective research. The GDPR also enforces in Europe individuals’ rights, such as portability right, the right to object to certain uses, and the right to withdraw. This means keeping patients updated about policies and uses foreseen in a registry.

Security measures regarding personal and sensitive data treatment in biomedical and epidemiological fields in many European countries are already well established and guarantee high-level protection of privacy. Confidentiality is insured on an operational level (pseudonymization, security measures). In this specific setting, the GDPR finds a solid environment to easily apply its provisions. To determine a country’s national guidelines, the relevant national Data Protection Commissioner’s Office should be contacted early on in the design of the registry, in relation with the Data Protection Officer of the legal entity responsible for the registry. It is crucial to ensure privacy of patients’ identities and in particular efficiently store patient identifiers. This includes pseudonymization of data to ensure that the patient is protected and double codification if data is shared. When considering data security, it is also important to formalise a policy document on data custodianship, access, and intellectual property. This should be set at an early stage via (1) an internal collaboration agreement between all participating legal entities (i.e., a consortium agreement) and (2) via a publicly accessible database access charter to inform potentially interested external users (e.g., BaMaRa (the French Rare Diseases Database) users’ charter [23] or I-DSD (International Disorders of Sex Development) standard operating protocol [24]). Data should only be accessed through secure channels and for specific purposes defined though a procedure that ensures traceability in order to be accountable for the usage of the data.

A crucial aspect of the registries is transparency. It is paramount that information about registry policies and operations are public and readily accessible to anyone interested. A good method to achieve transparency is: (a) the creation of a website of information about registry objectives and operations, registry information should be available in various media; (b) criteria on the capacity for sharing and exchanging data with other registries (national and international) supported by FAIR principles (quality); (c) access rules and duties for accessing parties (including clear intellectual property and exploitation policies, DTA (Data Transfer Agreement) rules etc.; (d) data security measures, traceability, informed consent, and its scope (e.g., possibility of secondary use of data)and patient’s rights implementation).

*Recommendation #6: Ensure compliance with (inter-) national and local ethical and legal requirements and on that basis, develop public policies for accessing maintaining and operating the registry.*

### 2.5. Sustainability

Sustainability is strictly connected to quality. High-quality RD registries have policies in place to ensure long-term sustainability and low quality registries are more likely to have been set up with few or no funds [25].

Very often, registries lack funding and resources and budgets are often exhausted by data collection and processing tasks alone. Few resources are left for maintaining the electronic infrastructure; performing security audits, quality controls, data analysis, interpretation; and the reporting and dissemination of important findings oriented towards appropriate medical and professional communities. In a number of cases, epidemiologists and statisticians are available on a temporary basis, or different persons are transitorily assigned to provide support on different occasions. Under these circumstances, a great deal of time has to be spent familiarizing personnel with the registry data, making temporary personnel far less useful. To the extent possible, funding should be ensured before the decision to implement a registry is made [10]. Very often, clinicians are likely to find data input too cumbersome, as it interferes with their routine work.

It is crucial to evaluate the budget early and ensure sufficient funding (and subsequent fund-raising efforts) for all activities related to the registry, to ensure its sustainability over a period that needs to be pre-determined. Expenses depend on the scope of the registry, costs for the underlying data capture tool, overall information system including costs for sensitive health data hosting and security audits, method of data collection, duration of data collection, number of participating centers, and process of data validation. Population-based registries, especially in the case of extensive data collection and/or long-term follow-up, are more expensive and time consuming to implement, compared with registries based on administrative and health data routinely collected by national, regional and local health authorities. In fact, population-based registries are demanding in terms of personnel and financial support, and therefore are very expensive, especially for the validation process of events, which should be homogeneous across countries so as to produce representative, accurate and comparable data.

The development and operation of a registry also requires long-term commitment. In many cases, the registry can take years to realize its full benefits; years for patient inclusions and years of longitudinal data collection are needed to answer the questions that the registry is designed to address.

Potential funding sources are pharma and the private sector at large; health care insurers; public authorities at the regional, national and European levels; patient organizations, professional associations. Registries can be funded from one or multiple sources. Shared funding mechanisms between public and private sources are becoming more common. Private-public partnerships have shown increasing interest in registries, to improve safety monitoring, compare effectiveness goals, and streamline the costs of drug or disposal development processes [26]. Multi-sponsor registries decrease the financial burden on each party, secure wider support, and limit financial risks when funding from one source fails [27].

*Recommendation #7: Ensure that the required budgets have been evaluated and that the registry is well resourced for a pre-defined period. For long-term sustainability, registries should seek funding from multiple, complementary sources.*

## 3. Data Source

### 3.1. Definition and Classification

Data source refers to the medical file where the actual data collection takes place and where all eligible patients are identified.

Data sources are classified as primary or secondary based on their relationship to the registry purpose. Primary data sources are collected for direct purposes of the registry [8] and are typically used when the data of interest is not available elsewhere or, if available, is unlikely to be of sufficient accuracy and reliability. Secondary data sources have already been collected for another purpose but are used by the investigator to examine a novel research question; in this case, information is transferred into the registry from existing databases. Examples of secondary data sources include medical records systems, administrative health insurance claims data, and death and birth records [6].

There are many advantages and disadvantages to using primary or secondary sources. The primary trade-offs relate to time, resources, and control of the collection of study variables. Using primary data sources, a researcher can control all aspects of the study methodology; all variables of interest can be measured. The creation of primary data sources requires wide consensus. They are in most cases costly and time consuming; however, they can also provide high-quality data. Secondary data sources are, on the other hand, less costly. They are easier to gather—providing that there is sufficient legal/regulatory clearance background and willingness of owners to share information. Secondary data sources may also cover a large geographic area and thus provide the ability to assess national trends; a disadvantage is that the data may not include all variables of interest [28]. Data from secondary sources are used either by automatic transfer into the registry or by linkage to other data sources to create a new, larger dataset for analysis. Data quality control before integration in the registry database is in both cases an important issue. It is worth emphasizing that the primary data source for one registry can act later as a secondary data source for another registry.

*Recommendation #8: The selection of primary data sources is critical for success of the registry; they provide data of higher quality than secondary data sources. Before incorporating a secondary data source into a registry, it is important to consider the legal and ethical feasibility of its incorporation and the potential impact of the data quality of the secondary data source on the overall data quality of the registry. Primary data sources are expensive and time consuming, thus, it is important to consider the possibility of reuse of existing data from secondary sources.*

### 3.2. Selection of Data Sources

Data sources are important windows for bias and their quality should be analyzed and known before the start of registry activities.

The most important aspect is their appropriate selection in terms of:

a) quantity:
  - representatively (geographic coverage (e.g., number of hospitals/disease expert centers in a particular region of the country),
  - size (e.g., number of patients enrolled for each hospitals) and
b) quality (completeness and capacity to identify patients with clear-cut diagnosis of interest).

With regard to data on rare events, a significant number of cases can be identified from a large population. Nevertheless, it is known that the ideal quality of cases identification for rare events is possible when one focuses on a limited population and conducts proper follow up and monitoring. Therefore, it is important to balance quantity versus quality, which also depends on the objectives of the registry. For a public health registry that is used to calculate incidence rates of diseases, it is essential to include all existing patient cases; therefore, quantity is of critical importance. On the other hand, for clinical registry, the importance of quality in case identification is greater than enrolment of all existing cases in the study. It is preferable to select data sources that guarantee a satisfactory level of quality, without the objective of exhaustive geographic coverage.

Data sources should also enroll patients who satisfy so-called inclusion and non-inclusion criteria, which are a set of conditions that a patient must meet to be eligible for inclusion in a registry. Generally included are descriptive characteristics (e.g., given molecular diagnosis, stage of disease) or demographic characteristics (e.g., age, gender, ethnicity) and geographic location (e.g., hospitals in a particular region of the country). Non-inclusion criteria, on the opposite hand, are those criteria that disqualify subjects from inclusion in the registry. Inclusion and non-inclusion criteria should therefore be defined as carefully as possible.

Particular attention should be paid to the ability of the data source to follow-up on included subjects. The so-called “lost to follow-up” is a critical issue because it may introduce a selection bias into the registry data. It is important to select data sources with potentially minimal loss of follow-up data.

*Recommendation #9: Provide clear-cut definition of inclusion and non-inclusion criteria. In terms of geographic coverage, when selecting data sources (e.g., hospitals), it is important to consider representativeness and ensure a satisfying level of quality in identifying all eligible patients (including the ability of proper follow-up).*

## 4. Data Elements, Case Report Form, Standardisations

### 4.1. Definition of Data Elements

A data element is a logical unit of data, which has a name, precise definition, and clear enumerated code values [29]. The data elements (DEs) that are included depends on the goal of the registry and it is important to carefully weigh the value of each data element to be included. Reducing the amount of data collected lowers costs, increases compliance, and improves the proportion of fields that are accurately completed. Too often, registries fail in their original purpose because information collection becomes too complex and unmanageable.

Planning linkages with other information systems during the developmental phases of the registry can eliminate redundant data elements from the system. It is often useful and time well spent to identify data elements that are essential or absolutely necessary and those that are “optional” or “desirable”, but may not be considered central to the key study hypothesis [30]. Inclusion of essential (or core) data elements in registries can enhance registry feasibility and sustainability by providing meaningful opportunity for sharing of data between registries.

Before deciding on data elements to be included in a registry, registry developers should gain an overview of the existing validated sources of data available and try to identify developed DEs that can be reused. Consultations with experts also ensure selection of appropriate DEs. Not only does this prevent problems that others have already resolved in defining data elements, but also facilitates data sharing and consistency. Another essential aspect is the dynamic annotation of the Case Report Form (CRF), which has an impact on the underlying database structure (coherence rules, automatic quality controls, conditional sections, and branching logics between DEs).

We identified a number of potential sources of DEs available worldwide:

- National Institute of Neurological Disorders and Stroke for neurological conditions (NINDS) [31].
- National Cancer Institute for Bioinformatics (NCICB) has developed the Common Data Elements (CDEs) for the cancer field [32]. NCICB stores the CDE in a relational database called CaDSR (Cancer Data Standards Repository) [33].
- The EPIRARE (European Platform for Rare Disease Registries) project [34] and RD Connect (integrated platform connecting databases, registries, biobanks and clinical bioinformatics for rare disease research) project [35] developed a minimum data set focused on rare disease registries.
- Translational Research in Europe for the Assessment and Treatment of Neuromuscular Disease for neuromuscular disorders (TREAT-NMD), including: (1) core data set for Duchenne Muscular Dystrophy (DMD) national registry [36]; (2) core dataset for international Facioscapular muscular dystrophy (FSHD) registry [37]; (3) core dataset for international Myotonic Dystrophy (DM1) registry [38]; (4) Spinal Muscular Atrophy (SMA) Registry Core Data [39].
- Patient-Reported Outcomes Measurement Information System (PROMIS) project created a web-based resource that features data field banks, case report form banks, and centralized access to computerized-adaptive testing for some measures [40].
- National Patient-Centered Clinical Research Network (PCORnet) Common Data Model (CDM) [41].
- Clinical Data Interchange Standards Consortium Operational Data Model (CDISC ODM) that develops data standards to streamline clinical research and enable connections to healthcare [42].
- Medical Data Models (MDM) portal is a meta-data registry for creating, analyzing, sharing and reusing medical forms. The portal, developed by the Institute of Medical Informatics, University of Muenster in Germany, maintains a repository of over 780,000 data elements [43].
- The U.S. National Library of Medicine (NLM) maintains a repository of common data elements, including relevant tools and resources, containing over 20,000 data elements [44].
- The French minimum data set for rare diseases (F-MDS-RD) is a minimum set of DEs agreed for mandatory collection and reporting at the national level [45].
- Joint Research Centre (JRC), together with The Directorate-General for Health and Food Safety (DG SANTE) released a Set of Common Data Elements for Rare Diseases Registration [46].

*Recommendation #10: The following steps are recommended for defining DEs:*

∙ *Determine what data are needed for the purpose(s) of the registry.*
∙ *Determine what information models and forms exist that can be reused.*
∙ *Determine what data are obtained from primary sources (requiring additional effort to collect) and from secondary sources (at the risk of lower data quality).*
∙ *Determine what data can be derived from other data, rather than being collected separately.*
∙ *Determine whether data can be collected and stored as a part of clinical care (thus becoming data from secondary source).*
∙ *Determine whether data can be fed back to assist clinical care.*

### 4.2. Establishment of Case Report Form (CRF) Questions

From a methodological point of view, it is important to distinguish the concept of Case Report Form (CRF) from DEs. The CRF is the interview questionnaire used to collect registry data and includes questions and data that the registry intends to collect from its patients. The CRF is a printed, optical or electronic document designed to record all of the protocol—required information to be reported to the sponsor on each trial subject [47].

**Figure 2:**
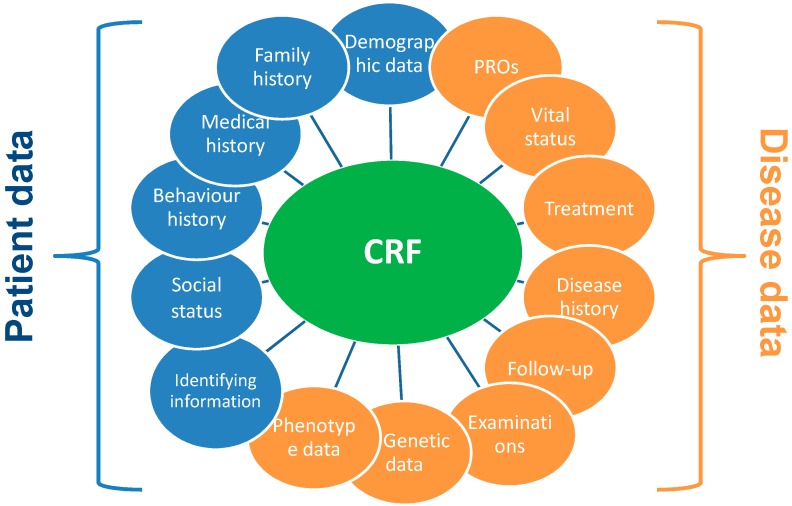
Examples of Case Report Form module.

It is recommended to establish and maintain a library of templates of standard CRF modules as they are time saving and cost-effective. A CRF Library is offered by the National Institute of Neurological Disorders and Stroke for neurological conditions (NINDS) Team [48]. Examples of CRF module are reported in Figure 2.

### 4.3. Standardisations

The standardisation of DEs is a critical part of developing a registry. The alignment of our DEs with standards (international or national) can be carried out through the use of a coding system, terminologies, vocabularies, and ontologies. The use of standards in a registry system is a quality indicator since this facilitates consistency, reuse and semantic interoperability. The Human Phenotype Ontology (HPO) and Orphanet Rare Disease Ontology (ORDO) are the most important standards used for phenotype descriptions and disease classification in the field of RD; both are promoted by International Rare Diseases Research Consortium (IRDiRC). Annotation of registry data with HPO and ORDO is a key starting point for ensuring interoperability [49,50] and complying with FAIR principles. Table 1 presents several international standards that are widely used in the health domain.

As mentioned, for each DE, a definition, identification, representation, and permissible values need to be specified. The definition is important to ensure that the meaning of the data element is maximally clarified (e.g., a check box for a disease may only be checked if presence of this disease has been professionally assessed). Identification is ideally done through the use of international standards, such as Logical Observation Identifiers Names and Codes (LOINC). Representation includes the specification of data types; for example, numerical data elements, dates, and coded data elements. Depending on the data type, permissible values can be specified, such as minimum and maximum values, or allowed values from a coding system.

*Recommendation #11: Ensure and promote the use of standards in the registry system, (a) for diseases classification such as ORPHANET and RD Ontology (ORDO) and (b) for phenotypes description (such as The Human Phenotype Ontology (HPO). Use of standards facilitates data interoperability.*

## 5. IT Infrastructure Complying with FAIR Principles

The IT infrastructure of a registry is used to store and manage all the data that the registry collects. The infrastructure should be easy to use by the registry managers, while at the same time secure and flexible to adjust to changing requirements. These requirements are seemingly in conflict and hard to implement. Therefore, it is important to select infrastructure in close collaboration with the IT department, registry users, and organisations’ legal counsels (to verify that the chosen solutions will meet the legal requirements set forth by the GDPR).

### 5.1. Infrastructure Selection and Implementation

The software chosen should closely match the registry’s requirements. It is tempting to choose a home-built system that can closely match requirements; however, a new system will be expensive and take a long time to implement, thus severely limiting registry’s ability to manage data. Therefore, it is ideal to implement an existing system that offers ready-to-use solutions. Several commercial and open source solutions are available for the same. Most of these systems offer easy customization of databases based on CRFs and data elements. Using a system that can import and export data and CRFs in a common standard format file such as CDISC ODM (Clinical Data Interchange Standards Consortium Operational Data Model) [42], XML (Extensible Markup Language) [51] or CSV (Comma Separated Values) [52] will provide a viable exit strategy if the chosen system is no longer sufficient for needs of the registry.

International standards such as ISO 27001, ISO 27002 [53] for information data security set requirements on the system’s security implementation. Legislations such as the GDPR [21] carry out stiff penalties in case of data leakage. Commercial solutions often come with appropriate certifications out of the box, but for systems that are not certified compliant out of the box, it is advisable to do a security assessment during the implementation phase.

To ensure continuous availability of the system, there should be a regular system maintenance plan that is maintained by the IT department. This plan should include the installation of relevant updates and security patches, monitoring of system errors and unusual patterns, and a backup strategy for data and systems consisting of a combination of regular full on-site and off-site encrypted backups. Especially for smaller registries, if the local IT department does not have the capacity to maintain the registry system, a hosted solution can be an alternative, provided that a data processor agreement compliant with the GDPR is present.

### 5.2. FAIR Data Principles

Registry managers aim for registered data to be used as extensively and efficiently as possible for the benefit of RD patients (within legal and ethical boundaries). Therefore, we consider not only the quality of the content of a registry (i.e., the values), but also the quality of how data are made available for wider use. Enabling efficient analysis of data across multiple registries and other data sources is powerful and necessary in the RD domain, but poses specific requirements on how data are made available. For instance, a researcher may wish to link known relations between treatments and symptoms to patient-reported symptoms in a vascular anomaly registry. It would be inefficient to re-collect information in the vascular anomaly registry that is already in Orphanet, and the linking would be very inefficient and error-prone if Orphanet and the registry use different terms to describe the same symptoms. Orphanet is one source of complementary information for a registry; a wealth of information is available across health data registries and repositories around the world. While for common diseases, ‘linkability’ of resources may be one of many priorities, for rare diseases it is a top priority. To efficiently answer a question across all relevant resources by computer, it is important that the sources use similar terminology for their values and data types. Data incompatibility has many drawbacks for the rare disease domain, where resources are scarce compared to the number of diseases. First, it creates an incentive to recollect data for every question again and again. Secondly, data researchers spend staggering amounts of time on repeatedly resolving incompatibilities and correcting errors, because of the guesswork involved in determining what data from different resources exactly mean. Thirdly, computers cannot aid in analysis across resources, because they depend on explicit, unambiguous definitions of data. To mitigate this problem, we recommend ‘data stewardship’ at the source and the application of the FAIR Guiding Principles for scientific data management and stewardship [54], which have recently been accepted as an IRDiRC recognized resource. ‘FAIRification’ requires guidance and interdisciplinary collaboration. Disease knowledge is required to describe what the data mean; information modelling expertise is required to encode data and make them available in a machine-readable form. The resulting FAIR data landscape allows analysis to be carried out on data at the source, even for sensitive data, while only sharing non-sensitive analysis results. The concept pertains to straightforward queries, such as ‘Which biobank contains samples of the same phenotype profile as those of my patient?’, to advanced computational analysis such as distributed machine learning for medical decisions support (e.g., doi:10.1016/j.ijrobp.2017.04.021).

*Recommendation #12: Involve registry users, the IT department and legal counsel in the selection process for IT infrastructure. Select an off-the-shelf solution that is open and provides data exports in FAIR and common open data formats. Conduct a security assessment as part of the implementation of your solution.*

## 6. Data Quality

Data are the product of the registry system, and are the basis for the production of information upon which decisions are made. The quality of data is defined by attributes such as completeness, accuracy, timeless, usefulness, interoperability, accessibility, and data security [13]. All these attributes are interrelated and it is important to define objectives for data quality and to find a good balance among them. Several methods exist to quantify data completeness, e.g., capture-recapture, and linkage with independent data sources. It is also important to state item completeness (% of missing values for each variable), detect duplicate records (% of duplicate records found in the whole database), check data accuracy (e.g., re-abstracting of data and evaluate % of agreement with external and independent data sources), and assess reproducibility of data collection (e.g., % of agreement among data collectors).

In order to address data quality issues, most registries have introduced quality assurance and quality control and quality assessment.

The quality process comprises of several aspects that can be categorized as quality assurance (activities undertaken before data collection to ensure that the data are of the highest possible quality at the time of collection), quality control (activities undertaken during and after data collection aimed at identifying and correcting sources of data errors) and quality assessment (process of quality evaluation of the whole registry system after having a consolidated database). Quality assurance, quality control and quality assessment can be expensive and their costs associated need to be recognised and included in the budget of the registry. In general, the higher level of completeness and accuracy are directly linked to the costs.

Recommendations for assuring data quality include: (1) Clear-cut inclusion and non-inclusion case criteria as well as target population should be defined; (2) analysis of sources of information and their capacity of providing valid information should be explored; (3) case selection and case ascertainment are the two most important questions to minimize selection bias; (4) control of duplicates and minimizing mistakes in the interpretation and diagnosis are important clues for quality; (5) errors in coding, data entry, and data transformation; data consistency across sites and over time; and intentional errors should be taken care of while using electronic forms. Personnel should act as data curators, and external audits should be conducted, among other mechanisms; (6) reliability and data accuracy should be frequently explored; (7) data completeness must be checked.

*Recommendation #13: In order to address data quality, introduce quality assurance and quality control activities at different levels. Monitor the start of data collection and regulatory data quality at the central level and locally (site monitoring). Produce regular data quality reports, including evaluation of different dimensions of data quality (e.g., completeness, accuracy, duplicate prevention and timeless).*

## 7. Quality Information

Analysis is an essential activity aimed at converting data into information. Collection of high-quality data is a prerequisite to producing correct and precise information, and a good statistical plan is needed to produce accurate results. In fact, the utility of collected data relies on the quality of the data analysis plan and the ability to interpret and disseminate the results [8].

Developing and checking transparent and consistent statistical analysis plans are activities related to quality assurance and quality control activities in the framework of quality information of a registry.

The data collected by a registry could be used to undertake descriptive or analytical studies. In all cases, it is important to develop a defined statistical analysis plan, describing statistical techniques to be used in order to address the objective(s) of the registry. In the framework of quality of a registry, descriptive analysis should be planned to ensure continuous monitoring. Epidemiological indicators should be defined and calculated in order to detect trends and evaluate impacts.

Registries should be also designed keeping in mind that they serve as sources of data for observational analytical studies, which aim to investigate the association of diseases to exposure. Information about patient characteristics (i.e., gender, age) or known risk factors (i.e., socioeconomic status) should be collected. Risk factors could also be associated with the investigated exposure representing confounders or effect modifiers, and they have to be considered in the statistical model [55]. Inclusion of variables regarding potential risk factors and accuracy of the collected information are key points in performing an analytical observational study and limiting the bias typical of this kind of study.

In order to facilitate a valid interpretation, the results of the analysis should be put into context through comparisons with external evidence. Incidence, prevalence, rates, and any outcome of interest should be compared to those obtained from other studies performed on different data sources, as long as they are comparable. Interpretation should also offer reasons as to why results are similar or different. In general, limiting the generalization of some types of results that await confirmation from other studies is a strategy that credits scientifically to the registry and, consequently, to its quality.

Interpretation of the analyses offers recommendations to stakeholders (i.e., patient organizations, health services researchers and providers, policy makers, drug agencies) to determine policy, update information about disease course, choose a treatment or intervention, and develop clinical practice guidelines.

Registries can provide such information through reports, which can be standardized according to registry-specific aims and objectives. Registries report descriptive data, taking care of the quality and validity of the information. The quality of a registry is closely associated with the validity of information that is provided through reports and other forms of dissemination such as bulletins, scientific papers and policy documents [25].

*Recommendation #14: Develop a plan for statistical analysis describing the techniques to be used in order to address the objective(s) of the registry. Ensure data dissemination to different stakeholders—registry participants, patients, general public, decision makers, and researchers.*

## 8. Documentation

Developing and maintaining transparent and adequate documentation on the registry is essential for ensuring quality and efficient operation. The key document is the manual, which should contain all types of explanations about registry functioning. Example of the list of documents contained in the manual of operations are: Policy rules and governance; IT tools document; security document; ethic rules; list of standard operating procedures; manual training; instructions for database users; data elements/dictionary/standardisations; form templates (e.g., consent forms and patient information notice); auxiliary tables (such as participating centers, declared user list) diagnosis tests, medical proofs; classifications; personnel functions and tasks; budgets; checklists of list of activities to be revised and marked after they have been developed by workers; nested research study protocols, quality assessment document containing all procedures, control mechanisms and methods for correct evaluation of the registry and their frequency. A standard operating procedure is an important part of this manual because it describes activities regarding data collection, cleaning, storing, monitoring, reviewing, and reporting. Each procedure has to be clearly written, workflow well defined, and involvement of each person clearly stated. In addition, a study protocol is a researcher’s blueprint that guides and governs all aspects of how a study is conducted. It also provides transparency in research, and improves the reproducibility and reusability of the research (complying with FAIR data standards), thereby potentially increasing the credibility and validity of a study’s findings [56]. The details of manuals vary, depending on the complexity and objective of the registry. Last but not least, documentation also supports transparency and follow up of all decisions made by the registry governance (agenda and minutes of meetings, contractual documents and possible amendments, etc.).

*Recommendation #15: Developing and maintaining transparent and adequate documentation is essential for ensuring the quality and efficient operation of the registry. Documentation details may vary, depending on the complexity of the registry.*

## 9. Training

Training is another important aspect of registry quality. Training is not only important for registry staff, but also for those of the healthcare units that provide the data.

The registry governance should provide a training plan to be provided on an ongoing basis, subjected to review and updates, and needs to carefully consider how extensive the training should be, who has to be trained (e.g., data providers, registry’s staff), what is the most appropriate way of training, and if any supporting material is needed. In multi-centre registries, a central training session is often the best way to ensure that all personnel is trained in a standardised manner.

Training is performed to provide knowledge on sources of information used in the registry; methods of data collection; methods of data input and checks; sensitization on tasks, respective obligations and responsibilities (e.g., carefully archiving patients’ informed consents); statistical analysis of data; linkage procedures; validation procedures; review of clinical events; standardization of procedures and methods for evaluation of quality of data; and most importantly to ensure that procedures adopted to generate high-quality data are implemented and understood in practice.

Training should be performed regularly or when updates or changes occur (changes to the data collection system; personnel, software for data entry and data element). Training activities lead to cost reduction over time.

*Recommendation #16: Ensure proper and systematic training of registry staff and data providers at all levels. Provide training in a systematic way prior registry access by new users, and when changes occur.*

## 10. Data Quality Audit

Data quality audits are independent systematic examinations of data that establish the extent to which registry data conform to predetermined standards or criteria.

Audits vary in scope, frequency and location, depending on the objectives of a registry and funding constraints.

An audit can be conducted on a random sample of participating sites (e.g., 5 to 20 percent of registry sites); and “for cause” (meaning only when there is an indication of a problem, such as one site being an outlier compared to most others) [8]. It can be conducted either on the whole set of data of the registry, or just for a select (random or systematic) sample of patients, using sampling techniques. Data should be collected in a manner that facilitates auditing.

The approach to auditing the quality of the data should reflect the most significant sources of error, with respect to the purpose of the registry.

The audit can assess enrolment of eligible patients, and quality of data (e.g., evaluation of data completeness or accuracy of diagnosis). It should also review records to ensure that all study procedures are being implemented and followed. Data audit can be performed by visiting the participating centers and comparing a sample of the data from the central registry database with the original source data. When an audit identifies serious problems, the leaders may decide to exclude that data from the overall results.

The audit can be internal or external. Internal audit is carried out by the registry staff, following a concrete plan and using specific indicators to assess the most significant sources of error. External audit is performed by external personnel, in accordance with pre-established criteria [6].

Subsequent to audits, the findings should be communicated to all staff, indicating the areas for improvement and highlighting good practice in order to facilitate learning.

*Recommendation #17: Have in place an audit system, which includes defined triggers that initialize the process.*

## 11. Conclusions

The development of RD registries is seeing considerably growth, as the field deals with a scarcity of patients, and the need to gather cohort data to enable research, better define natural history and epidemiology, facilitate clinical research studies, and assess healthcare standards.

In this article, we discussed the essential aspects influencing registry quality. A list of recommendations was reported in Table 2 and elaborated upon.

Recommendations made by experts were focused on, which included the establishment of registry governance, identification of correct data sources, specification of data elements, case report forms, standardisations, construction of a IT infrastructure complying with FAIR principles, production of data quality, and dissemination of quality information. Other topics, such as developing adequate documentation, training of staff and providing data quality audit, are also considered essential for improving registry quality.

It is hoped that these recommendations can help registry owners and curators facilitate self-quality assessments to operate and maintain high-quality registries. Future work in this field includes developing a toolkit based on process indicators to check the quality of existing registries, and provide a score and feedback on aspects of the registry that require improvement.

In conclusion, in the field of RD registries, there is a need for coordination between ongoing initiatives at the national and international levels. At the national level, we recommend the development of a centralized, public, national, “registry-as-a-service” platform, which will guarantee access to highly-trained staff on all topics mentioned in the article, foster the standardisation of registries, allow economy of cost and time for setting up new registries, allow interlinking of key data sources on different diseases, and increase the capacity to develop cooperation at the regional level (Europe, in this case). At the national level, platforms for RD registries in Europe have been collaborating to create a centralized European Union framework on patient registries that offer data sharing, reduce duplication of efforts and costs, facilitate validation of results, enable engagement with experts and the patient community, and overcome the “rare disease problem” in terms of cohort size, powering trials, and finding confirmatory cases.

## Figures and Tables

**Table 1:** Existing international standards used in the biomedical field.

Area	Standard	Developer	Website
Disease	ICD	WHO	http://www.who.int/classifications/icd/en/
	ORDO	ORPHANET	http://www.orphadata.org/cgi-bin/inc/ordo_orphanet.inc.php/
Medical Nomenclature	SNOMED	SNOMED International	https://www.snomed.org/snomed-ct
Phenotypic terms	HPO	Charité Berlin and with the Monarch Initiative	http://human-phenotype-ontology.github.io/
Rare Clinical signs	ICHPT	IRDIRC	http://www.irdirc.org/activities/current-activities/ichpt/
Medical and administrative data	HL7		http://www.hl7.org/implement/standards/index.cfm?ref=nav
Device	GMDN	GMDN Maintainance Agency	https://www.gmdnagency.org/About/Database
Drugs	ATC DDD index	WHO	https://www.whocc.no/atc_ddd_index/
	MEDDRA	International Conference on Harmonisation (ICH)	www.meddra.org
Adverse reactions	WHO Art	WHO	https://www.who-umc.org/vigibase/services/learn-more-about-who-art/
Functioning	ICF	WHO	http://www.who.int/classifications/icf/en/
Primary care	ICPC-2	WHO	http://www.who.int/classifications/icd/adaptations/icpc2/en/
Genes, genetic disorders	OMIM	McKusick-Nathans Institute of Genetic Medicine, Johns Hopkins University School of Medicine	https://www.ncbi.nlm.nih.gov/omim
Laboratory and clinical observations	LOINC	Regenstrief Institute	https://loinc.org/

**Table 2:** List of recommendations for Improving the Quality of Rare Disease Registries.

Topics	Recommendations
Registry definition	*#1 RD registries are organised information system, based on observational study designs with one or several predefined purposes and rules with a long-term perspective. We recommend that RD registries should not be limited by geographical area but have an international vision. In the field of RD, there is a need to identify as many patients as possible, and significant number of cases could be identified from a large population. Moreover, RD registries should be created and used as a data source for the purpose of performing subsequent or additional studies related to research, patient care or public health monitoring.*
Registry classification	*#2 In order to fulfil the expected objectives, the taxonomic position of the registry should be clearly defined. It can be classified into several categories according to different criteria (e.g., geographical coverage, diseases coverage, type of sponsors, methods used for data collection etc.).*
Governance	*#3 Define clear objectives and design the structure of the database consequently. Often registries start with a single purpose, but can evolve into multi-purposes, addressing the interests of other collaborators and stakeholders. In RD registries objectives should be expanded but it is preferable to prioritise and define them clearly.* *#4 Engage with all relevant stakeholders, especially patient representatives at an early stage in the implementation of a registry.* *#5 Establish a good registry team with clear role and responsibilities for all staff working members in proportion with the registry’s size, ambition and objectives. Identify a registry leader, domain expert, IT expert and ELSI expert from the start. Either employ an IT person within your registry or setup a long-term partnership with a commercial company.* *#6 Ensure compliance with (inter-) national and local ethical and legal requirements and on that basis develop public policies for accessing maintaining and operating the registry.* *#7 Ensure that the start-up required budgets have been evaluated and that the registry is well resourced for a pre-defined period. For long-term sustainability, registries should to seek funding from multiple, complementary sources.*
Data Source	*#8 The selection of primary data sources are critical for the success of the registry; they provide data of higher quality than secondary data sources; before incorporating a secondary data source into a registry, it is important to consider the legal and ethical feasibility of its incorporation and the potential impact of the data quality of the secondary data source on the overall data quality of the registry. Primary data sources are expensive and time consuming, so you should consider the possibility of reuse of existing data from secondary sources.* *#9 Provide clear-cut definition of the inclusion and non-inclusion criteria. In terms of geographical coverage, when selecting data sources (e.g., hospitals), it is important to consider the representativeness and ensure a satisfying level of quality in identifying all eligible patients (including the ability of proper follow-up).*
Data Elements, Case Report Form, Standardisations	*#10 The following steps are recommended for defining Data Elements:* *Determine what data are needed for the purpose(s) of the registry.* *Determine what information models and forms exist that can be reused.* *Determine what data are obtained from primary sources (requiring additional effort to collect) and from secondary sources (at the risk of lower data quality).* *Determine what data can be derived from other data, rather than being collected separately.* *Determine whether data can be collected and stored as part of routine clinical care (thus becoming data from secondary source).* *Determine whether data can be fed back to assist clinical care.* *#11 Ensure and promote the use of standards in the registry system (a) for diseases classification such as ORPHANET and RD Ontology (ORDO) and (b) for phenotypes description (such as The Human Phenotype Ontology (HPO). Use of standards facilitates the data interoperability.*
IT Infrastructure complying with FAIR principles	*#12 Involve registry users, the IT department and legal counsel in the selection process for the IT infrastructure. Select an off-the-shelf solution that is open and provides data exports in FAIR and common open data formats. Conduct a security assessment as part of the implementation track of your solution.*
Data Quality	*#13 In order to address data quality, introduce quality assurance and quality control activities at different levels. Monitor from the start of data collection and then regulatory data quality at central level and locally (site monitoring); produce regular data quality reports including evaluation of different dimensions of data quality (e.g., completeness, accuracy, duplicate prevention and timeless).*
Quality information	*#14 Develop a plan for statistical analysis describing the statistical techniques to be used in order to address the objective(s) of the registry; ensure data dissemination to different stakeholders: registry participants’, patients, general public, decision makers and researchers.*
Documentation	*#15 Developing and maintaining transparent and adequate documentation is essential for ensuring the quality and efficient operation of the registry. The detail of documentation may vary from registry to registry depending on the complexity of the registry.*
Training	*#16 Ensureproper and systematic training at all levels, addressed to registry staff and data providers. Provide training in a systematic way prior registry access by new users and when changes occur.*
Data quality audit	*#17 Have an audit system including defined triggers initializing audit processes.*

## References

[1] The Council of the European Union (2009). Council Recommendation of 8 June 2009 on an Action in the Field of Rare Diseases (2009/C 151/02). Off. J. Eur. Union.

[2] (2011). Directive 2011/24/EU of the European Parliament and of the Council of 9 March 2011 on the Application of Patients’ Rights in Cross-Border Healthcare. Off. J. Eur. Union.

[3] Orphanet Report Series-Rare Disease Registries in Europe—May 2018. http://www.orpha.net/orphacom/cahiers/docs/GB/Registries.pdf.

[4] RD-Connect. https://rd-connect.eu/.

[5] Guidelines for Data Sources and Quality for RD Registries in Europe. http://www.epirare.eu/del.html.

[6] Zaletel M., Kralj M. (2015). Methodological Guidelines and Recommendations for Efficient and Rational Governance of Patient Registries.

[7] (2013). Neurological Registry Best Practice Guidelines—Complete Document. Can. J. Neurol. Sci..

[8] Gliklich R., Dreyer N., Leavy M. (2014). Registries for Evaluating Patient Outcomes: A User’s Guide.

[9] Brooke E.M. (1974). The Current and Future Use of Registers in Health Information Systems.

[10] Solomon D.J., Henry R.C., Hogan J.G., Van Amburg G.H., Taylor J. (1991). Evaluation and implementation of public health registries. Public Health Rep..

[11] U.S. Department of Health and Human Services (2011). Postmarketing Studies and Clinical Trials Implementation of Section 505(o)(3) of the Federal Food, Drug and Cosmetic Act. Silver Spring: FDA Guidance for Industry.

[12] Santoro M., Coi A., Di Paola M.L., Bianucci A.M., Gainotti S., Mollo E., Taruscio D., Vittozzi L., Bianchi F. (2015). Rare disease registries classification and characterization: A data mining approach. Public Health Genom..

[13] Kodra Y., Posada de la Paz M., Coi A., Santoro M., Bianchi F., Ahmed F., Rubinstein Y.R., Weinbach J., Taruscio D. (2017). Data Quality in Rare Diseases Registries. Adv. Exp. Med. Biol..

[14] James S., Rao S.V., Granger C.B. (2015). Registry-based randomized clinical trials—A new clinical trial paradigm. Nat. Rev. Cardiol..

[15] Li G., Sajobi T.T., Menon B.K., Korngut L., Lowerison M., James M., Wilton S.B., Williamson T., Gill S., Drogos L.L. (2016). Registry-based randomized controlled trials—What are the advantages, challenges, and areas for future research?. J. Clin. Epidemiol..

[16] EURORDIS-NORD-CORD Joint Declaration of 10 Key Principles for Rare Disease Patient Registries. https://www.eurordis.org/content/eurordis-nord-cord-release-joint-declaration-10-key-principles-rare-disease-patient-registries.

[17] Abma T.A., Broerse J.E. (2010). Patient participation as dialogue: Setting research agendas. Health Exp..

[18] European Bioinformatics Institute (EMBL-EBI). https://www.ebi.ac.uk/training/online/course/biomedical-data-ethical-legal-and-social-implicati/biomedical-data-collection-use-and-shari-2.

[19] Sariyar M., Schluender I., Smee C., Suhr S. (2015). Sharing and Reuse of Sensitive Data and Samples: Supporting Researchers in Identifying Ethical and Legal Requirements. Biopreserv. Biobank..

[20] Mascalzoni D., Dove E.S., Rubinstein Y., Dawkins H.J., Kole A., McCormack P., Woods S., Riess O., Schaefer F., Lochmüller H. (2016). International Charter of principles for sharing bio-specimens and data. Eur. J. Hum. Genet..

[21] Regulation (EU) 2016/679 of the European Parliament and of the Council of 27 April 2016 on the Protection of Natural Persons with Regard to the Processing of Personal Data and on the Free Movement of Such Data, and Repealing Directive 95/46/EC (General Data Protection Regulation) (Text with EEA Relevance). http://eur-lex.europa.eu/eli/reg/2016/679/oj.

[22] Gainotti S., Turner C., Woods S., Kole A., McCormack P., Lochmüller H., Riess O., Straub V., Posada M., Taruscio D. (2016). Improving the informed consent process in international collaborative rare disease research: Effective consent for effective research. Eur. J. Hum. Genet..

[23] The French Rare Diseases Database (BaMaRa) Terms of Use of BaMaRa. http://www.bndmr.fr/participer/mode-autonome/cgu/.

[24] The International Disorders of Sex Development Registry I-DSD Registry Protocol for a Research Database. https://www.gla.ac.uk/media/media_247070_en.pdf.

[25] Coi A., Santoro M., Villaverde-Hueso A., Di Paola M.L., Gainotti S., Taruscio D., De La Paz M.P., Bianchi F. (2016). The quality of rare disease registries: Evaluation and characterization. Public Health Genom..

[26] Lyratzopoulos G., Patrick H., Campbell B. (2008). Registers needed for new interventional procedures. Lancet.

[27] De Groot S., van der Linden N., Franken M.G., Blommestein H.M., Leeneman B., van Rooijen E., Koos van der Hoeven J.J., Wouters M.W., Westgeest H.M., Uyl-de Groot C.A. (2017). Balancing the Optimal and the Feasible: A Practical Guide for Setting Up Patient Registries for the Collection of Real-World Data for Health Care Decision Making Based on Dutch Experiences. Value Health.

[28] Carlson M.D.A., Morrison R.S. (2009). Study Design, Precision, and Validity in Observational Studies. J. Palliat. Med..

[29] National Institute of Neurological Disorders and Stroke for Neurological Conditions (NINDS) Common Data Elements. https://commondataelements.ninds.nih.gov/Glossary.aspx?term=Data+Element.

[30] Saczynski J.S., McManus D.D., Goldberg R.J. (2013). Commonly used data-collection approaches in clinical research. Am. J. Med..

[31] National Institute of Neurological Disorders and Stroke for Neurological Conditions (NINDS) Common Data Elements. https://commondataelements.ninds.nih.gov/ProjReview.aspx#tab=Introduction.

[32] Nadkarni P.M., Brandt C.A. (2006). The common data elements for cancer research: Remarks on functions and structure. Methods Inf. Med..

[33] National Cancer Institute. https://wiki.nci.nih.gov/display/caDSR/caDSR+Database.

[34] EPIRARE (European Platform for Rare Disease Registries). http://www.epirare.eu/_down/del/D9.3_ProposalforCDE_FINAL.pdf.

[35] RD-Connect. http://rd-connect.eu/rdcon/files/RD-Connect_CDEs_May_2016.pdf.

[36] TREAT-NMD Neuromuscular Network 2013. http://www.treat-nmd.eu/downloads/file/registries_toolkit/DMD_core_dataset_May2013.pdf.

[37] TREAT-NMD Neuromuscular Network 2011. http://www.treat-nmd.eu/downloads/file/registries_toolkit/FSH_core_dataset_May2011.pdf).

[38] TREAT-NMD Neuromuscular Network 2009. http://www.treat-nmd.eu/downloads/file/registries_toolkit/DM1_core_dataset_August2009.pdf.

[39] TREAT-NMD Neuromuscular Network 2014. http://www.treat-nmd.eu/downloads/file/registries_toolkit/SMA_core_dataset_March2014.pdf.

[40] Health Measures. http://www.healthmeasures.net/explore-measurement-systems/promis.

[41] PCORnet, the National Patient-Centered Clinical Research Network. http://www.pcornet.org/pcornet-common-data-model/.

[42] Clinical Data Interchange Standards Consortium. https://www.cdisc.org/standards/data-exchange/odm.

[43] Medical Data Models Portal. http://medical-data-models.org/.

[44] NIHCommon Data Element (CDE) Resource Portal. https://www.nlm.nih.gov/cde/.

[45] Choquet R., Maaroufi M., de Carrara A., Messiaen C., Luigi E., Landais P. (2015). A methodology for a minimum data set for rare diseases to support national centers of excellence for healthcare and research. J. Am. Med. Inform. Assoc..

[46] European Commission Joint Research Centre (JRC). https://ec.europa.eu/jrc/sites/jrcsh/files/set-data-elements-rare-diseases-registration.pdf.

[47] U.S Food and Drug Administration (2018). E6(R2) Good Clinical Practice: Integrated Addendum to ICH E6(R1) Guidance for Industry.

[48] National Institute of Neurological Disorders and Stroke for Neurological Conditions (NINDS) CRF Library. https://commondataelements.ninds.nih.gov/CRF.aspx.

[49] Maaroufi M., Choquet R., Landais P., Jaulent M.C. (2014). Formalizing mappings to optimise automated schema alignement: Application to rare diseases. Stud. Health Technol. Inform..

[50] Maaroufi M., Choquet R., Landais P., Jaulent M. (2015). Toward data integration automation for the French rare disease registry. AIMIA Annu. Symp. Proc..

[51] Extensible Markup Language (XML). https://www.w3.org/XML/.

[52] Comma Separated Values (CSV). https://tools.ietf.org/html/rfc4180.

[53] International Organization for Standardization (2013). ISO/IEC 27000 Family—Information Security Management Systems.

[54] Wilkinson M.D., Dumontier M., Aalbersberg I.J., Appleton G., Axton M., Baak A., Blomberg N., Boiten J.W., da Silva Santos L.B., Bourne P.E. (2016). The FAIR Guiding Principles for scientific data management and stewardship. Sci. Data.

[55] Rothman K., Greenland S., Lash T.L. (2008). Modern Epidemiology.

[56] The European Network of Centres for Pharmacoepidemiology and Pharmacovigilance (ENCePP) (2012). Guide on Methodological Standards in Pharmacoepidemiology (Revision 1).

